# Multidisciplinary Prospective Study of Mother-to-Child Chikungunya Virus Infections on the Island of La Réunion

**DOI:** 10.1371/journal.pmed.0050060

**Published:** 2008-03-18

**Authors:** Patrick Gérardin, Georges Barau, Alain Michault, Marc Bintner, Hanitra Randrianaivo, Ghassan Choker, Yann Lenglet, Yasmina Touret, Anne Bouveret, Philippe Grivard, Karin Le Roux, Séverine Blanc, Isabelle Schuffenecker, Thérèse Couderc, Fernando Arenzana-Seisdedos, Marc Lecuit, Pierre-Yves Robillard

**Affiliations:** 1 Neonatal and Pediatric Intensive Care Unit, Pôle Mère-Enfant, Groupe Hospitalier Sud-Réunion, Saint-Pierre, La Réunion, France; 2 Center of Clinical Investigation–Clinical Epidemiology (CIC-EC, INSERM), Saint-Pierre, La Réunion, France; 3 Department of Gynecology and Obstetrics, Pôle Mère-Enfant, Groupe Hospitalier Sud-Réunion, Saint-Pierre, La Réunion, France; 4 Department of Microbiology, Pôle des Sciences Biologiques, Groupe Hospitalier Sud-Réunion, Saint-Pierre, La Réunion, France; 5 Department of Neuroradiology, Pôle de Radiologie, Groupe Hospitalier Sud-Réunion, Saint-Pierre, La Réunion, France; 6 Fetal Medicine and Fetopathology Unit, Pôle Mère-Enfant, Groupe Hospitalier Sud-Réunion, Saint-Pierre, La Réunion, France; 7 National Reference Center for Arboviruses, Institut Pasteur, Lyon, France; 8 Institut Pasteur, Microorganisms and Host Barriers Group, F-75015, Paris, France; 9 Inserm, Equipe Avenir U604, F-75015, Paris, France; 10 Institut Pasteur, Molecular Viral Pathogenesis Unit, F-75015, Paris, France; 11 Inserm U819, F-75015, Paris, France; 12 Department of Infectious Diseases and Tropical Medicine, Necker-Pasteur Center for Infectious Diseases, Hôpital Necker-Enfants Malades, Assistance Publique-Hôpitaux de Paris, F-75015, Paris, France; United States Department of Defense, United States of America

## Abstract

**Background:**

An outbreak of chikungunya virus affected over one-third of the population of La Réunion Island between March 2005 and December 2006. In June 2005, we identified the first case of mother-to-child chikungunya virus transmission at the Groupe Hospitalier Sud-Réunion level-3 maternity department. The goal of this prospective study was to characterize the epidemiological, clinical, biological, and radiological features and outcomes of all the cases of vertically transmitted chikungunya infections recorded at our institution during this outbreak.

**Methods and Findings:**

Over 22 mo, 7,504 women delivered 7,629 viable neonates; 678 (9.0%) of these parturient women were infected (positive RT-PCR or IgM serology) during antepartum, and 61 (0.8%) in pre- or intrapartum. With the exception of three early fetal deaths, vertical transmission was exclusively observed in near-term deliveries (median duration of gestation: 38 wk, range 35–40 wk) in the context of intrapartum viremia (19 cases of vertical transmission out of 39 women with intrapartum viremia, prevalence rate 0.25%, vertical transmission rate 48.7%). Cesarean section had no protective effect on transmission. All infected neonates were asymptomatic at birth, and median onset of neonatal disease was 4 d (range 3–7 d). Pain, prostration, and fever were present in 100% of cases and thrombocytopenia in 89%. Severe illness was observed in ten cases (52.6%) and mainly consisted of encephalopathy (*n* = 9; 90%). These nine children had pathologic MRI findings (brain swelling, *n* = 9; cerebral hemorrhages, *n* = 2), and four evolved towards persistent disabilities.

**Conclusions:**

Mother-to-child chikungunya virus transmission is frequent in the context of intrapartum maternal viremia, and often leads to severe neonatal infection. Chikungunya represents a substantial risk for neonates born to viremic parturients that should be taken into account by clinicians and public health authorities in the event of a chikungunya outbreak.

## Abstract 2

In a prospective study on the island of La Réunion, Marc Lecuit and colleagues find frequent transmission of Chikungunya virus by viremic mothers giving birth during an outbreak, resulting in serious infant illness.

## Editors' Summary

**Background.:**

Chikungunya virus, an emerging infectious agent that is transmitted by day-biting mosquitoes, was first isolated from a patient in Tanzania in the early 1950s. Since then, major outbreaks of chikungunya fever have occurred throughout sub-Saharan Africa and in Southeast Asia, India, and the Western Pacific, usually at intervals of about 7–8 years. The virus causes fever, rash, severe joint and muscle pains, and sometimes arthritis (joint inflammation). These symptoms develop within 3–7 days of being bitten by an infected mosquito. Most people recover fully within a few weeks, but joint pain can sometimes continue for years. There is no treatment for chikungunya fever, but the symptoms can be eased with anti-inflammatory drugs. Preventative measures include covering arms and legs and using insecticides to avoid insect bites and depriving the mosquitoes of their breeding sites by draining standing water from man-made containers near human dwellings.

**Why Was This Study Done?:**

In 2005, chikungunya fever appeared for the first time on several islands in the Indian Ocean. On La Réunion Island, the disease affected 300,000 people—more than one-third of the population—between March 2005 and December 2006. In June 2005, clinicians identified the first case of mother-to-child chikungunya virus transmission (vertical transmission). Public-health officials and clinicians need to know more about how often vertical transmission occurs and its clinical implications to help them prepare for future chikungunya fever outbreaks. In this study, the researchers identify and characterize all the cases of vertical chikungunya virus transmission that occurred at the largest hospital on La Réunion Island during the 2005–6 outbreak.

**What Did the Researchers Do and Find?:**

The researchers enrolled all 7,504 women who gave birth at the hospital during the outbreak and their 7,629 children into their study. They then used “RT-PCR” (which detects the genome of virus particles during an active infection) and “IgM serology” (which looks for an immune response to recent infection) to determine which women had been infected with chikungunya virus during their pregnancy. 678 of the new mothers had been infected sometime between conception and a week before delivery, 22 mothers had been infected between 7 and 3 days before delivery, and 39 had been infected 2 days either side of delivery (the “intrapartum” period). Except for three early fetal deaths that were associated with chikungunya virus infections, vertical transmission was seen only in babies born to mothers infected with the virus intrapartum. 19 of the babies born to these women were infected with the virus—a vertical transmission rate of nearly 50%. The women who transmitted the virus to their offspring had more virus in their placenta than those who did not transmit the infection. Delivery by emergency cesarean section did not prevent transmission. All the infected babies were born healthy but developed fever, weakness, and pain within 3–7 days. In many of them, the number of platelets (clot-forming particles) in their blood also dropped dramatically. Ten babies became seriously ill—nine of them developed brain swelling; two had bleeding into their brain. Four children had lasting disabilities at the end of the study.

**What Do These Findings Mean?:**

These findings show that mother-to-child transmission of chikungunya virus occurs frequently when women are infected with the virus at the time of delivery and that newborn children infected by this route can become very ill. Although these results do not find that cesarean section reduces infection rates, 90% of cesarean sections involving infected infants were performed urgently, rather than planned. The study also provides no information about whether delaying delivery, provided that no fetal distress is observed, until the mother's viral load has decreased might be beneficial. More studies are needed to provide a complete description of both the short-term and long-term effects of chikungunya virus infection in newborn babies, but it is clear that clinicians should monitor babies exposed to chikungunya virus during delivery for a week after their birth. Most importantly, clinicians and public-health officials will need to take account of the threat that the chikungunya virus poses to newborn children whenever and wherever it emerges.

**Additional Information.:**

Please access these Web sites via the online version of this summary at http://dx.doi.org/10.1371/journal.pmed.0050060.

- Read the related *PLoS Medicine* Perspective article
- The World Health Organization provides information about chikungunya fever and a brief description of the recent chikungunya outbreak in the Indian Ocean (in English, French, Spanish, Arabic, Chinese, and Russian)
- The US Centers for Disease Control and Prevention has a fact sheet on chikungunya fever
- The UK Health Protection Agency also provides information about chikungunya virus, including news on recent outbreaks
- The French Institut de Veille Sanitaire (Institute for Public Health Surveillance) has a Web page on chikungunya (in French)
- The Institut Pasteur has a Web page on chikungunya research (in French and English)

## Introduction

The chikungunya virus (CHIKV) is an enveloped, positive-strand RNA alphavirus belonging to the *Togaviridae* family and transmitted by *Aedes* mosquito bites [1]. It causes a dengue-like illness, characterized by fever, rash, painful myalgia, and arthralgia, and sometimes arthritis [2]. It was first isolated by R.W. Ross in 1952 in the Newala district of Tanzania [3]. Its current geographic distribution covers sub-Saharan Africa, Southeast Asia, India, and the Western Pacific where numerous outbreaks have been reported [4–8]. In these areas, upsurges of re-emergence occur at intervals of 7 to 20 years [9].

Since the end of 2004, CHIKV has emerged in the Southwestern Indian Ocean islands. Between January and March 2005, over 5,000 cases were reported in the Comoros. Later in 2005, the virus spread to other islands, including Mayotte, Seychelles, La Réunion, and Mauritius [9]. In La Réunion Island, a French overseas department (total population: 787,836), the first declared case was observed in Saint-Pierre (southern area of the island) in the beginning of March 2005 among people returning from the Comoros [10]. The transmission was moderate until the rainy season, which started in December in 2005 and was associated with an epidemic of unprecedented magnitude (300,000 cumulative cases on December 30, 2006), with a peak incidence reached on the fifth week of 2006 (over 45,000 cases). No other known arboviral disease was associated with this chikungunya outbreak.

Aedes albopictus was identified as the only vector of a principally urban transmission in La Réunion [11]. During this outbreak, severe or complicated forms of chikungunya were reported in adult patients, including encephalopathy and hemorrhagic fever, which frequently occurred in the context of chronic diseases or underlying conditions such as diabetes mellitus, chronic obstructive pulmonary disease, ischemic heart disease, chronic renal failure, or alcoholic hepatopathy [10,12].

In June 2005, we identified the first case of mother-to-child chikungunya virus transmission [13]. We thus conducted a prospective study in order to characterize the epidemiological, clinical, biological, and radiological features and outcomes of all the cases of mother-to-child chikungunya infections recorded at our institution during this outbreak.

## Methods

### Study Location and Participants

Our prospective study took place in the level-3 public maternity department of the Groupe Hospitalier Sud-Réunion (GHSR), the largest hospital on the island (1,300 beds, standard care comparable to that available in Europe, 4,300 deliveries per year for a population of 300,000 inhabitants; 80% of the births within the area, the remaining taking place in a private level-1 maternity department). The level-3 maternity department of the GHSR is well insulated and air-conditioned, and at the peak of the chikungunya outbreak, blood-derived products were imported from a chikungunya-free area, minimizing the risk of nosocomial CHIKV transmission, whether by mosquito bite or blood-derived products.

We enrolled all parturient women and their offspring admitted at the maternity department between 1 June 2005 (date of the first chikungunya infection observed in the course of a pregnancy) and 30 December 2006 (date of delivery of the last pregnant woman infected with chikungunya). For the use of the data, oral consent was obtained from each patient or a first-degree relative, as the investigations were carried out under the standard care procedure for this new disease, in accordance with the recommendations of the Committee for Clinical Research of the GHSR. In France, written consent is mandatory only if the medical treatments or the products used are not standard for the diagnosis, treatment, or monitoring (art. 88-II, law 2004–806, *Journal Officiel*, 08/11/2004; art. 31-I, law 2006–450, *Journal Officiel*, 04/19/2006). The information was given in French or in Creole with the help of a translator when necessary.

### Data Collection and Screening

Midwives collected full obstetric history from mandatory maternity booklets and additional questioning in the framework of our daily epidemiologic perinatal survey [14].

As the positive predictive value for chikungunya infection of the association of fever with rash or arthralgia during the outbreak was higher than 95% [15], and the rate of clinically silent cases below 5% (Gérardin et al., unpublished data), serological screening for chikungunya was performed in all pregnant women who had presented with these clinical signs during the course of their pregnancy but were not diagnosed with chikungunya infection prior to their pregnancy. The delay for IgM seroconversion was 5 to 7 d after the onset of symptoms, whereas that for IgG was up to 15 d [16]. CHIKV-specific IgM antibodies were detected by capture ELISA [17] and CHIKV-specific IgG antibodies by ELISA [18] developed at the Centre National de Référence des Arbovirus (Institut Pasteur, Lyon, France) and automated with an EtiMax 3000 apparatus (DiaSorin, Italy). Each serum sample was assayed in a well coated with culture supernatant of a CHIKV-infected cell line (wAg) and in a well coated with culture supernatant of the corresponding uninfected cell line (woAg). A reference serum (ref serum) was tested in triplicate on each 96-well microplate. The results were expressed in arbitrary units to normalize the inter-assay variability and were calculated as follows: (OD serum sample wAg − DO serum sample woAg) / [(Σ^1−3^ (OD ref serum wAg − OD ref serum woA) / 3] × 100. The cut-off values for IgMs and IgGs were determined from a series of 30 negative sera collected in La Réunion Island before the epidemic started. Positive and negative control sera were added on each 96-well microplate.

For symptomatic women, whether presenting for examination at the outpatient clinic or for hospitalization at the maternity ward, a one-step TaqMan real time quantitative RT-PCR was performed in serum samples using the Light Cycler 2.0 system (Roche Diagnostics). Oligonucleotide primers amplified a conserved coding region of glycoprotein E1 [19]. A positive homologous internal control was included for monitoring RNA extraction and detecting false negatives. A synthetic RNA was used as an external standard for CHIKV quantification. The assay sensitivity for serum specimen was 350 genome copies per milliliter [20]. The window of positive real-time quantitative RT-PCR ranged from the day of the onset of symptoms (day 0) to day 5.

The timing of chikungunya maternal infection was determined after delivery, based on clinical criteria as well as RT-PCR and IgM/IgG serology results (see Table 1). In the absence of evidence of earlier infection, maternal chikungunya was classified as antepartum in 678 women whose clinical signs had occurred between conception and the week preceding labor, and diagnosed by RT-PCR performed prospectively for outpatients, or retrospectively at delivery by detection of IgM antibodies. Maternal chikungunya was classified as prepartum in 22 women with symptoms lasting between day −7 and day −3 before delivery and diagnosed by RT-PCR (or IgM seroconversion when not available), and as intrapartum in 39 women with symptoms between day −2 and day 2 around delivery and concomitant positive RT-PCR (or IgM seroconversion when not available).

**Table 1:**
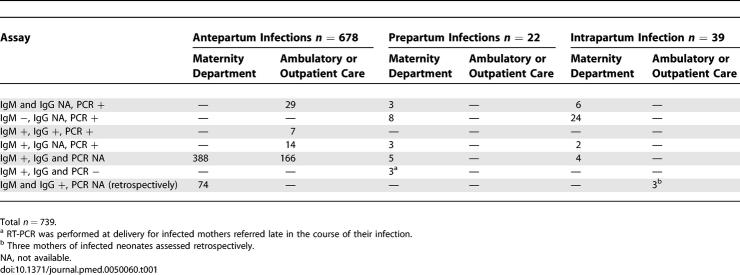
Serological and/or RT-PCR Evidence for Chikungunya Diagnosis in Ante-, Pre-, and Intrapartum Infections/Exposures, Groupe Hospitalier Sud-Réunion, Saint-Pierre, La Réunion, France, 2005–2006 Outbreak: Mothers

Exposed neonates were screened by IgM and IgG assays at days 0, 7, and 15. For 57 out of 62 neonates born from a mother viremic in pre- or intrapartum, a RT-PCR test was also performed on serum at day 0 and/or during the first week of life upon clinical indication. The RT-PCR and serological results for the diagnosis of chikungunya are provided for 591 out of 749 neonates in Table 2.

**Table 2:**
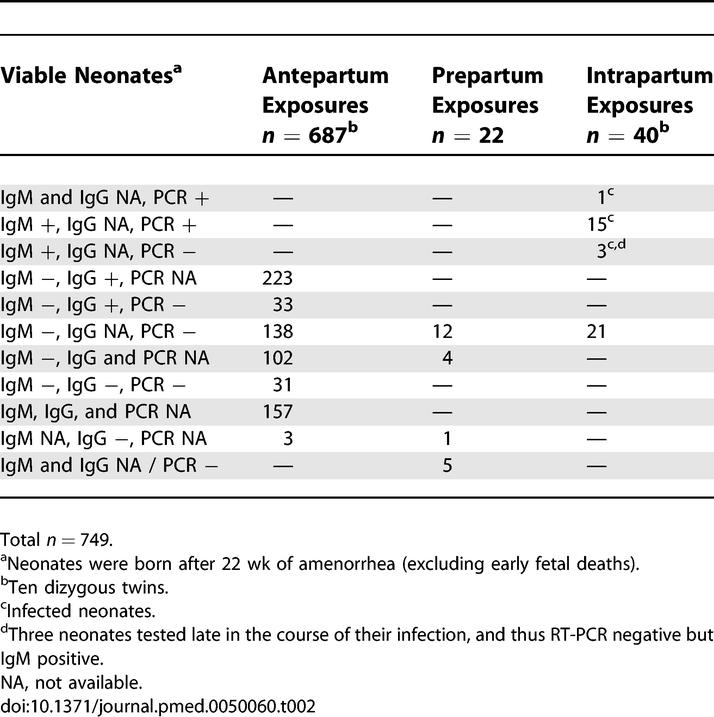
Serological and/or RT-PCR Evidence for Chikungunya Diagnosis in Ante-, Pre-, and Intrapartum Infections/Exposures, Groupe Hospitalier Sud-Réunion, Saint-Pierre, La Réunion, France, 2005–2006 Outbreak: Viable Neonates

Clinical features associated with neonatal infections were collected during the stay in the neonatal department (28 beds, ten for neonatal intensive care unit [NICU]; 350 admissions per year). For neonates, the chikungunya was considered as a vertical mother-to-child transmission when symptoms occurred during the first week of life, in the absence of evidence of mosquito bite. For mothers and neonates, cases not confirmed by RT-PCR or IgM serology were excluded. Clinical variables included the mode of delivery, fetal heart rate, gestational age, birth weight, 5-min Apgar score, delay between birth and onset of symptoms (defining the incubation period), fever, cutaneous and rheumatologic signs (rash, petechiae, and joint edema), hematological parameters (total blood cell count and hemoglobin), and blood biochemical parameters (sodium, glucose, calcium, urea, creatinine, and liver enzymes). In case of a severe thrombocytopenia (platelet count < 100,000/mm^3^), blood coagulation tests (prothrombin time, partial thromboplastin time, fibrinogen, and D-dimers) were performed to diagnose disseminated intravascular coagulation (DIC) syndrome.

The brain MRI protocol included T1-weighted imaging (T1WI) before and after the intravenous infusion of a gadolinium-based contrast agent, T2-weighted imaging (T2WI), and diffusion-weighted imaging (DWI) with apparent diffusion coefficient (ADC) maps. Neurological follow-up of infected newborns after discharge from the neonatal department was performed using a standardized neurological test [21].

### Statistical Analyses

Monthly cumulative incidence rates, *i.e*., attack rates, were measured as the ratio of the pregnant women newly infected per month divided by the sum of the women delivered during the same month plus those anticipated to deliver in the eight following months. Monthly prevalence rates were calculated for the parturient women as the ratio of the number of parturients infected during their pregnancy and delivered in the month over the number of parturients in the given month.

In order to determine the obstetrical and neonatal characteristics of mother-to-child transmission of CHIKV, a case-control study was conducted, after exclusion of early antepartum fetal deaths (APFDs) before 22 wk. Clinical and biological continuous parameters were compared with the Mann-Whitney test. The rates of fetal heart decelerations, cesareans sections (C-sections), and neonatal asphyxia (defined as 5-min Apgar score < 7) for the cases of vertical transmission of CHIKV and for a randomly selected control group of uninfected neonates of the same gestational age range (born from uninfected mothers and hospitalized at the same time in the neonatal care unit) were compared using Chi-square or Fisher exact tests, when appropriate. Two controls per case were included. All analyses were computed in Stata (Stata Statistical Software: release 9; StataCorp. 2005). A *p*-value < 0.05 was considered statistically significant.

## Results

**Figure 1:**
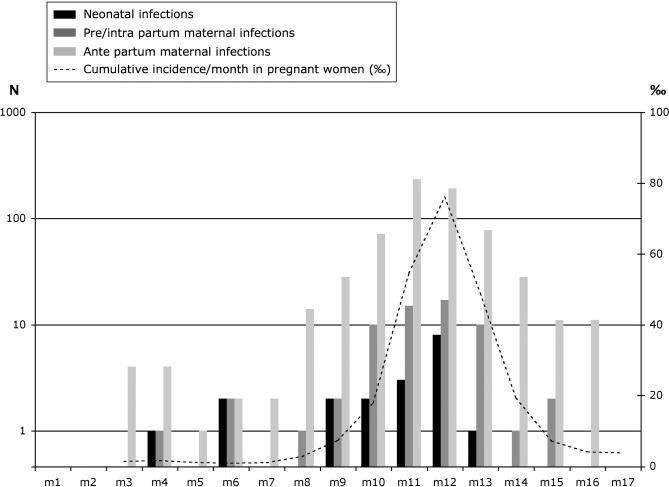
Monthly Evolution of Neonatal, Maternal Pre- and Intrapartum, and Antepartum Chikungunya Cases between March 2005 (m1) and June 2006 (m16), First Sud-Réunion Outbreak, in Saint-Pierre

During this 22-mo long survey (March 2005 to December 2006), 7,504 consecutive women delivered 7,629 viable neonates at the level-3 GHSR maternity department whilst about 2,000 births occurred in the level-1 maternity department. The monthly evolution of the cumulative incidence of maternal chikungunya infections during pregnancy and that of neonatal cases observed in the GHSR level-3 maternity are presented in Figure 1. The first reported antepartum case occurred in May 2005 (third month after the beginning of the outbreak, m3) and the last in June 2006 (m16). During the first 9 mo of the outbreak (cold season in the Southern hemisphere), the attack rate among pregnant women was below 1% and the prevalence rate among parturient women was below 5%, owing to a sporadic transmission (fewer than ten cases per week). At m10, the hot and rainy season (austral summer) started, and the attack rates increased sharply during m10–m12 in the general population (45,000 new cases during the first week of February 2006). For pregnant women, the incidence peaked in January 2006 (m11) with an attack rate of 8.3% (95% confidence interval [CI] 7.4%–9.3%). Among parturient women, the peak of prevalence was delayed to m15 with a 27.5% reported rate. Between m13 and m16 the attack rates decreased sharply to 0.4% (95% CI 0.15%–0.6%) and then prevalence rates felt dramatically to reach only 0.4% in December 2006 (m22), marking the end of our survey.

In total, of the 7,504 parturient women, 739 (9.8%) reported a history of chikungunya during pregnancy, including 678 (9.0%) in the antepartum period (i.e., more than a week before delivery), and 61 (0.8%) in pre- (*n* = 22) or intrapartum (*n* = 39) (i.e., symptoms between day −7 and day −3, or day −2 and day 2 around delivery, respectively) (Table 1). Chikungunya maternal infection (i.e., fetal exposure to maternal blood-borne chikungunya) distribution was uniform throughout pregnancy (median onset of maternal infection: 25 wk; interquartile range [IQR] 16–33; range 0–41; mode 36), and no significant breakdown or peak in infection during pregnancy was observed. During the same period, fewer than 20 cases of antepartum maternal infection, and no pre- or intrapartum maternal or neonatal infection, were reported in the level-1 private maternity department.

The prevalence rate of APFD after 22 wk for the Sud-Réunion area throughout the chikungunya outbreak did not differ from previous annual rates available since 2001 (0.9% between March 2005 and December 2006, 0.5% in 2001, 0.7% in 2002, 1% in 2003, and 1.2% in 2004). Among the 678 women infected antepartum and whose pregnancy was monitored from the second trimester, nine APFD were reported after 22 wk, and none was attributable to CHIKV infection (negative CHIKV RT-PCR for amniotic fluid, fetal brain, and serum). Among the seven early APFDs that were reported before 22 wk, only three were attributable to CHIKV infection: the three pregnant women were viremic (positive serum CHIKV RT-PCR) at the onset of symptoms (12 wk 4d, 15 wk, and 15 wk 5d), and APFD was observed around two weeks later [22]. For these three APFDs, the amniotic fluid collected by amniocenteses before fetal demise was RT-PCR positive. CHIKV RNA was detected in the placenta and in the fetal brain for two. These three women were no longer viremic at the time of miscarriage, excluding a postmortem contamination of the fetus from the maternal blood. Of the four remaining early APFDs, CHIKV RNA was not amplified from fetal samples.

None of the children of the 678 pregnant women infected antepartum had detectable IgM at birth (Table 2), and mother-transferred IgG, surveyed in 70 infants, cleared progressively: 3% of the infants were IgG negative at 3 mo of age, 43% at 6 mo, and 81% at 9 mo. On follow-up, the absence of IgM at 3 months in these children ensured that they had not been infected.

Most pre- and intrapartum maternal cases were diagnosed by RT-PCR, and only three prepartum-infected women referred late in the course of the infection were RT-PCR negative (Table 1). Among the 61 women who presented with ongoing chikungunya infection in the setting of delivery (22 in pre- and 39 in intrapartum) (Table 1), 19—all with an intrapartum infection—transmitted the chikungunya to their offspring (vertical mother-to-child transmission rate for intrapartum infections: 19/39, 48.7%) (Table 2). All mother-to-child transmissions occurred in the context of near-term deliveries (median length of gestation: 38 wk, range 35–40 wk). During the labor of the 61 women experiencing viremia around term, none received blood-derived product before the section of the umbilical cord. Forty-six fetuses (75%) exhibited deep spikes or late decelerations on fetal heart monitoring. These abnormalities were seen in the cases of vertical transmission (14/19) as well as in its absence (32/42), and were more likely to occur in CHIKV-infected women than that from uninfected control neonates (see Methods) (73.7% versus 39.5%, *p* = 0.01, Table 3). Of the 61 women experiencing a CHIKV infection in the setting of delivery, the rate of C-section was 42.6%, of which 69.2% were performed because of acute fetal distress. This rate significantly exceeded the 17.4% overall C-section rate observed in our center (*p* ≤ 0.001) [14]. However, it was not significantly different than that of randomly selected parturients whose neonates were hospitalized at the same time in the neonatal care unit (Table 3). Importantly, C-section had no influence on mother-to-child viral transmission, either for intrapartum infections (C-section rates in infected versus uninfected neonates: 48.7% versus 52.9%, NS), or globally for pre-/intrapartum infections (unpublished data).

**Table 3:**
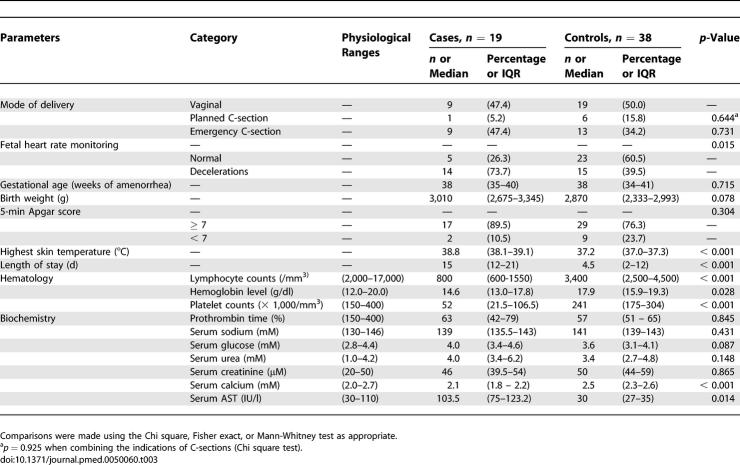
Admission Characteristics of 19 Neonatal Cases of Chikungunya Mother-to-Child Infection and of 38 Unexposed Controls Treated in the Neonatal Care Unit of Groupe Hospitalier Sud-Réunion, Saint-Pierre, La Réunion, France, 2005–2006 Outbreak

The viral load in the placentas of seven of the 19 transmitters (mean ± standard deviation [SD]: 42,000 ± 20,167 copies/mg of tissue) was significantly higher than that in 13 placentas of the nontransmitters (mean ± SD: 10,742 ± 8,182 copies/mg, Mann Whitney test, *p* = 0.021). Among the 19 transmitters, one gave birth to dizygous twins: one neonate remained uninfected, whereas the other became infected.

Of the 19 neonates who developed a vertical chikungunya infection in the setting of maternal viremia, all were exposed in intrapartum and none was symptomatic at birth nor had a history of mosquito bite. In none of the 16 neonates diagnosed by RT-PCR (mean viral load 250 million copies/ml of plasma) was the CHIKV genome detected at day 1 (viral load < 350 copies/ml). The median onset of neonatal disease (defining the incubation time) was 4 d after birth (range 3–7 d). Among the 19 infected neonates, ten were breast-fed. Maternal milk, tested for 20/33 breast-feeding viremic women, was always RT-PCR negative. No infection was reported over the same time among hospitalized neonates born from non-viremic mothers.

In our series, the main clinical features at presentation were fever, poor feeding, and pain, observed in all infected infants (100%) and evidenced by need for constant analgesic treatment and enteric feeding. Rheumatic and cutaneous signs also constituted major associated clinical manifestations: distal joint edema (15/19, 78.9%), petechiae (9/19, 47.3 %), or polymorphous rubella-like (10/19, 52.6%) or roseola-like exanthema (7/19, 36.8%).

The most frequent physiological abnormality was thrombocytopenia (17/19, 89.4%), associated with a mild elongation of prothrombin time (*n* = 6) and DIC (*n* = 4). Severe thrombocytopenia (9/19, 47.3 %) led to empiric administration of hydrocortisone (*n* = 8), gamma globulins (*n* = 1), or both (*n* = 2), and platelet infusions (*n* = 4) with the aim of avoiding massive hemorrhages, known to be life-threatening in the context of dengue hemorrhagic fever (DHF). Other biological abnormalities included lymphopenia (13/19, 68.4%), which preceded the onset of clinical signs in half of the cases, a mild increase of AST level (10/19, 52.6%), and a moderate to severe hypocalcemia (9/19, 47.3 %).

The admission characteristics of the 19 neonates treated at our center for vertical chikungunya infection are presented and compared to unexposed controls in Table 3.

Severe neonatal disease was observed in ten cases (52.6%) and mainly consisted of encephalopathy (*n* = 9), or hemorrhagic fever (*n* = 1). Cerebrospinal fluid (CSF), collected for the nine cases with encephalopathy, was RT-PCR positive for CHIKV in five cases (mean viral load 184,000 copies/ml of CSF), but normal for chemistry and cytology (except for three cases with more than 3,500 RBCs/mm^3^, one of which was in the context of DIC). Of the ten severe cases, eight required mechanical ventilation (median duration: 7 d). Shock/hypovolemia was observed in six cases; each displayed a hyperkinetic profile on echocardiography, and four required the use of vasoactive amines (median duration 2 d). DIC, reported in four cases, was complicated by transient brain hemorrhages in two—namely, scattered parenchymal petechiae in both hemispheres with additional bleeding, cerebellar hematoma in one, and hematemesis in the other. Indicators of severity at admission were a normal skin temperature (*p* = 0.008), a low prothrombin time (*p* = 0.002), and a low platelet count (*p* = 0.035) (presented in detail in Table 4).

**Table 4:**
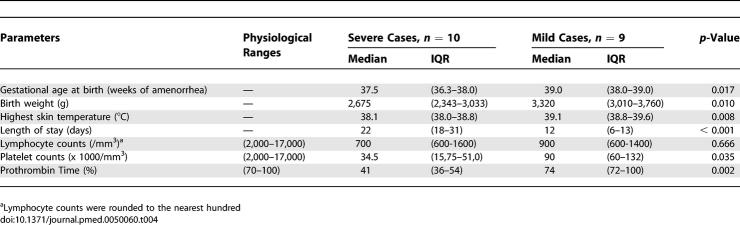
Factors Associated with Severe Disease in the 19 Neonates Treated in the Neonatal Intensive Care Unit of Groupe Hospitalier Sud-Réunion, Saint-Pierre, La Réunion, France, for a Chikungunya Mother-to-Child Infection, 2005–2006 Outbreak

**Figure 2:**
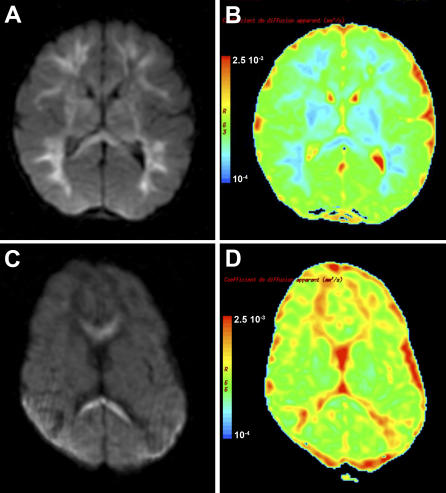
Representative MRI Findings in Neonatal Chikungunya Encephalopathy Cases Observed during the First Chikungunya Outbreak in Saint-Pierre, La Réunion, June 2005 to December 2006 (A) Day 6 (child A), scattered hyperintensity signals of the white matter involving the corpus callosum, the frontal and parietal lobes on diffusion-weighted imaging (DWI). (B) Day 6 (child A), scattered reduction of the Apparent Diffusion Coefficient (ADC) of the white matter involving the *corpus callosum*, the frontal and parietal lobes on ADC mapping (in blue). (C) Day 21 (child B), scattered and characteristic hypo-intensity signals of the white matter involving frontal and parietal lobes on DWI (Note that the *corpus callosum* remains in hyper-signal). (D) Day 21 (child B), scattered increase of the ADC of the white matter involving the *corpus callosum*, the frontal and parietal lobes on ADC mapping (in red).

The most distinctive MRI abnormalities observed in the course of neonatal chikungunya encephalopathy are shown in Figure 2. At the acute phase stage (6–15 d after onset of disease), MRI revealed unremarkable features on T1WI and T2WI, but several scattered and sometimes hyperintense signals of the supratentorial white matter were observed (Figure 2A), involving the corpus callosum and the frontal, parietal, and temporal lobes on DWI evocative of an early cytotoxic edema. Similar images were observed in the six cases for which MRI was obtained at the acute phase, and were associated with a marked reduction of the ADC (Figure 2B), compatible with parenchymal ischemia. Early evolution in the subacute phase (15–45 d after onset of disease) showed that the areas of hyperintense signals were replaced by areas of very low intensity on DWI (Figure 2C) in favor of an evolution into a vasogenic edema. These images were observed in all nine cases of encephalopathy and were associated with a concomitant increase of the ADC (Figure 2D) showing the reperfusion of low-output areas. They regressed in seven neonates but evolved towards cavitations and subcortical atrophy in two (unpublished data). No infants died, even those with severe shock or massive hemorrhage.

For the nine neonates with encephalopathy and brain swelling images, neurological sequelae were assessed upon discharge and throughout a 16- to 24-month follow-up. Four evolved towards persistent disabilities: one developed cerebral palsy with ataxia and blindness following extensive white matter degeneration for which a CACH/VWM (childhood ataxia with central nervous system hypomyelination/leucoencephalopathy with vanishing white matter) syndrome was ruled out (no mutation on *EIF2B*); three had ocular and behavioral or postural deficiencies (dysconjugate gaze, *n* = 3; language delay, *n* = 2; axial hypotonia, *n* = 1). These four children had multiple seizures during their original neonatal hospitalization and one of them still requires anticonvulsants at this writing. For the ten other neonatal chikungunya infections of our series, the clinical status and the brain MRI, assessed on regular follow-up, were considered normal.

## Discussion

Here we report the epidemiological, clinical, biological, and radiological features and outcomes of maternal-fetal transmission of CHIKV infection in an outbreak that occurred on the island of La Réunion, France. We find that mother-to-child CHIKV transmission is almost exclusively observed in the context of intrapartum maternal viremia, and often leads to severe neonatal infection. CHIKV thus represents a significant risk for neonates born from viremic parturients that must be taken into account by clinicians and public health authorities in the event of a chikungunya outbreak.

Given that the GHSR level-3 maternity department receives all pregnant women at risk in Sud-Réunion and that the level-1 maternity department declared a very low rate of maternal CHIKV infections because of a lower exposure of its population [15], and no neonatal infection, one may assume that the incidence and the prevalence rates calculated in our survey represent a good approximation of the true burden of chikungunya, both in pregnant women and in neonates, for the southern area of La Réunion Island.

We demonstrate here that mother-to-child transmission of CHIKV is relatively rare, since 2.5% (19/749) of exposed neonates became infected, although 10% (749/7,629) of the neonates were exposed during pregnancy, leading to an overall prevalence of maternal-fetal infections after 22 wk of 0.25% (19/7,629). In contrast, during the delivery period, the rate of transmission for viremic women was close to 50%, highlighting the intrapartum period as the critical time for transmission to the neonate. To our knowledge, the youngest child ever diagnosed with a chikungunya infection before this outbreak was a 21-d-old infant who was infected by a mosquito bite [23]. None of the 19 infected neonates recorded in this study, all of whom were born to mothers with an intrapartum infection, presented evidence of mosquito bites. Moreover, no infection was reported among concomitantly hospitalized neonates born to mothers without infection, emphasizing the absence of exposure to mosquito bites or contaminated blood-derived products in the maternity department. Together, these data provide evidence that these neonatal infections were the consequence of mother-to-child transmission complicating intrapartum maternal viremia. The absence of detectable neonatal viremia at day 1 of life is consistent with intrapartum maternal–fetal viral transmission.

Our preliminary investigations on the pathophysiology of maternal–fetal CHIKV transmission easily ruled out neonatal contamination through the genital tract, because gastric aspirations and nasal swabs were RT-PCR negative and C-sections had no protective effect on viral transmission [24]. Even though the mean viral load of placentas from infected neonates was significantly higher than that of uninfected neonates (*p* = 0.021), immunofluorescence labeling with anti-chikungunya antibody did not allow the detection of infected cells in any of these placentas (unpublished data), nor in an animal model of maternal-fetal chikungunya infection [25]. In addition, RT-PCR performed on isolated cells obtained from mechanical dissociation of these placentas was consistently negative (unpublished data), a result favoring the hypothesis of placental passive contamination by maternal blood-borne free virus particles rather than an actual placental infection. This hypothesis fits our observation that, in contrast to many in vitro-cultured cell types, the syncytiotrophoblast is not permissive to CHIKV [25].

Taken together, these observations suggest that the placental barrier is effective during antepartum in preventing maternal–fetal CHIKV transmission, as it was documented in only three cases (3/678, 0.4%) [22]. In contrast, viral maternal–neonatal transmission is frequently observed in viremic mothers around the term of pregnancy, when the highly viremic maternal blood (mean viral load 1.5 million copies/ml of plasma) can be in contact with placental barrier breaches resulting from uterine contractions during labor (vertical transmission rate of 48.7% in neonates exposed during labor). The higher viral load measured in placentas from infected neonates is thus most likely a consequence of a higher maternal viremia, which could therefore be predictive of the likelihood of transmission. We could not confirm this hypothesis, because of a lack of concomitant maternal blood samples corresponding to these placentas.

In areas where the *Aedes* vector is present, painful arthralgia complicating a dengue-like disease are strongly evocative of chikungunya [26]. In neonates, although evidence is limited, we estimate that painful arthralgia were present in 78%–100% of our cases, associated with distal joint edema and persistent prostration. Cutaneous signs, namely polymorphous rubelliform and roseoliform exanthema, were common findings in maternal–fetal neonatal chikungunya infections. These observations are consistent with previous chikungunya descriptions that reported possible maculopapular rash in adults [2,27] and in children [23,28] and that illustrate the frequency of skin manifestations in human arboviral diseases [29,30].

A low lymphocyte count was a common finding in neonatal chikungunya infection (nearly 70% of the cases), justifying the surveillance of white blood cell counts in neonates in our maternity hospital during the outbreak. However, as for dengue fever, lymphopenia is not a marker of severity in neonatal chikungunya [31]. In contrast, the intensity of thrombocytopenia, observed in 89% of infected neonates, was associated with severe neonatal disease and led to the administration of multiple supportive interventions including steroids and gammaglobulins to avoid bleeding complications, although their benefit have been demonstrated neither in DHF [32,33] nor in chikungunya. In our series, severe central nervous system (CNS) hemorrhages, namely scattered cerebrum-parenchyma petechiae, were seldom observed, but always in a clinical context of DIC syndrome, highlighting the rarity of chikungunya per se as a cause of hemorrhagic fever [28,34]. These clinical data are consistent with those reported by Ramful et al., who recently published a clinical description of the 38 neonatal cases reported to the local health authorities throughout the outbreak in La Réunion Island [28]. These investigators reported possible cardiac involvement, which appears to have been mainly characterized by coronary artery dilatations (six out of the 16 cases who underwent cardiovascular investigations). However, given its retrospective design and the fact that it focused on infected mother–child pairs but not on the whole population of uninfected and infected mothers throughout pregnancy, this study could not evaluate the modalities of neonatal transmission nor assess the transmission rate.

Cases of encephalopathy have been reported in adults during the La Réunion Island outbreak [12]. These cases were not associated with CSF, EEG, and MRI abnormalities (Lemant et al. unpublished data). In contrast, CNS involvement was observed in one-third of our neonatal cases, and was associated with a massive brain swelling as evidenced by MRI. This swelling may account for a transiently increased permeability of the blood–brain barrier without virus-induced damage of the CNS (RT-PCR–positive CSF but normal cytology and protein level in CSF samples, and absence of gadolinium-contrast enhancement), although a direct virus-induced encephalitis cannot be ruled out. In neonates and some adults, notably those with underlying conditions, CHIKV thus appears to exhibit a neurotropism, to our knowledge not yet described. Other neurotropic arboviruses include other alphavirus species, such as Eastern equine encephalitis virus and Venezuelan equine encephalitis virus, which are both associated with severe encephalitis in adults, as well as flavivirus family members such as West Nile virus, which can be associated with potentially severe CNS disease, notably in neonates and immunosuppressed patients [35]. The most distinctive lesions of chikungunya-associated neonatal encephalopathy were exclusively located in the white matter and consisted of areas of reversible diffusion restriction, a pattern classically associated with transient ischemia with cytotoxic edema that does not imply neuronal death [36]. These clinical and neuroradiological findings are in agreement with our current investigations in an animal model for CHIKV infection [25]. In this experimental model, viral infection of the CNS is mainly detected at the meningeal and ependymal levels rather than in the brain parenchyma, and no viral-associated neuropathology is detected.

Even when complicated by shock or DIC syndrome with gastrointestinal or cerebral bleeding, severe neonatal chikungunya infection—whether consisting of an encephalopathy or mimicking DHF—was never fatal in our series. This is a more favorable prognosis than that reported for neonatal DHF, which still carries high lethality in NICUs [37,38]. Nevertheless, the neurological outcome of chikungunya encephalopathy was unpredictable and exhibited a wide range of possible sequelae, ranging from mild ocular, behavioral, or postural deficiencies to severe cerebral palsy with extensive white matter damage mimicking a CACH/VWM syndrome [39].

Our identification of the first vertically transmitted CHIKV infection led to the rapid design of a prospective study aimed at determining the epidemiology and the modalities of maternal-fetal transmission of CHIKV. This study was facilitated by the existence of a birth register, adequate laboratory facilities, and interdisciplinary collaborations at GHSR. However, given the magnitude of the outbreak and our unpreparedness to deal with such an infectious disease crisis, some aspects of vertically transmitted CHIKV infection remain unknown, such as pregnancy status as a factor influencing the course of CHIKV infection and the putative preventive effect on neonatal transmission of postponing delivery until after resolution of maternal viremia. In addition, the limited size of our series of neonatal cases prevented a more exhaustive description of the disease in this age group. Last, the absence so far of long-term follow-up of infected neonates does not allow yet the assessment of CHIKV-associated long-term disabilities, such as learning or cognitive impairments, apart from those already noted at 16–24 mo follow-up.

In conclusion, maternal–fetal chikungunya transmission is rare at the population level, exceptional in antepartum, but is frequent in the setting of maternal viremia around term, and is associated with severe neonatal complications. Given our observations that maternal–fetal transmission almost invariably occurs in the setting of maternal viremia concomitant with delivery, and that C-section does not prevent CHIKV vertical transmission, we do not recommend the systematic performance of C-sections on infected mothers to reduce the risk of viral transmission, but to closely monitor viremic parturients and deliver them in maternity facilities with adequate obstetric and neonatal care. However, our study does not allow the conclusion that elective C-section of infected mothers without fetal distress would not protect from vertical transmission. In the absence of fetal distress, the putative preventive effect on fetal transmission of postponing delivery until resolution of maternal viremia remains to be determined. As already shown for other maternal–fetal transmitted viruses including hepatitis B virus and West Nile virus, viral neutralization in exposed neonates may constitute a useful approach to prevent neonatal infection. Our study did confirm, at a large scale, the kinetics of transplacental mother-transferred CHIKV-specific IgG antibodies, which lasted longer than 6 mo for more than 50% of La Réunion Island infants [18], as previously demonstrated for Thai children [40]. Whether these passively transferred antibodies exhibit a neutralizing and protective activity remains to be determined.

Given our observation that exposed neonates are not symptomatic at birth but become ill before day 7, we recommend retaining them in maternity care for a week with serial measurements of white blood cell and platelet counts, and transferring them to the NICU as soon as they become symptomatic, lymphopenic, or moderately thrombocytopenic.

As a consequence of the recent dramatically increased distribution of the chikungunya vector around the globe [11], CHIKV has the potential to cause massive outbreaks in the future. It may indeed not only re-emerge in areas where it has already been isolated, such as the Indian subcontinent (currently afflicted by a considerable outbreak), but also emerge in geographical areas where it is not known to have circulated previously (i.e., in nonimmune populations) [41] such as the American and European continents; this possibility is illustrated by the recent detection of CHIKV transmission in the Emilia-Romagna region of Italy [42]. Public health agencies and clinicians should be aware of the existence of maternal–fetal transmission of chikungunya and be prepared to diagnose and treat this severe neonatal infection.

## Supporting Information

Alternative Language Abstract S1 Abstract Translated into French by Patrick Gérardin (22 KB DOC)

## Abbreviations

ADC: apparent diffusion coefficient
APFD: antepartum fetal death
CHIKV: chikungunya virus
CI: confidence interval
CNS: central nervous system
CSF: cerebrospinal fluid
DHF: dengue hemorrhagic fever
DIC: disseminated intravascular coagulation
DWI: diffusion-weighted imaging
GHSR: Groupe Hospitalier Sud-Réunion
m[number]: month post-outbreak
IQR: interquartile range
NICU: neonatal intensive care unit
T1WI: T1-weighted imaging
