# Supplementary material for: Multidisciplinary Prospective Study of Mother-to-Child Chikungunya Virus Infections on the Island of La Réunion
Source: PLoS Med. 2008 Mar 18;5(3):e60. doi: 10.1371/journal.pmed.0050060 (PMC2267812; doi:10.1371/journal.pmed.0050060)
Supplement: Supplementary file 1 [file pmed.0050060.sd001.doc]

Étude prospective multidisciplinaire des infections materno-néonatales à virus Chikungunya diagnostiquées au Groupe Hospitalier Sud–Réunion entre mars 2005 et décembre 2006

***Contexte*** : Entre mars 2005 et décembre 2006, une épidémie d’infections à virus Chikungunya (CHIKV) a touché plus d'un tiers de la population de l'île de la Réunion. En juin 2005, nous avons identifié le premier cas de transmission mère-enfant du CHIKV dans la maternité de niveau-3 du Groupe Hospitalier Sud-Réunion.

***Méthodes et résultats*** : Nous avons mené une étude prospective afin de caractériser l’épidémiologie et les aspects cliniques, biologiques et radiologiques ainsi que l’évolution de tous les cas d’infections à Chikungunya transmises verticalement enregistrés dans notre hôpital au cours de l’épidémie. Le diagnostic d’infection à CHIKV était confirmé soit par une RT-PCR positive, soit par une sérologie IgM positive. En 22 mois, 7504 femmes enceintes ont accouché de 7629 nouveau-nés viables, parmi lesquelles 678 femmes (9,0 %) ont étés infectées en antepartum à distance de l’accouchement, et 61 (0,8 %) ont été infectées en prepartum (virémie positive entre J-7 et J-2 avant l’accouchement) ou intrapartum (virémie positive entre J-2 et J+2 autour de l’accouchement). À l’exception de trois fausses couches précoces, la transmission verticale du CHIKV a exclusivement été observée en cas d’accouchement au voisinage du terme (durée médiane de grossesse : 38 semaines d’aménorrhée (SA), étendue 35-40 SA), dans un contexte de virémie intrapartum (19 cas sur 39 expositions, taux de prévalence : 0,25%, taux de transmission verticale : 48,7%). La césarienne n’avait pas d’effet protecteur sur cette transmission. Tous les nouveau-nés infectés étaient asymptomatiques à la naissance et leurs premiers symptômes sont apparus après une durée médiane de 4 jours (étendue 3-7 j). La triade douleur, prostration, fièvre était présente dans 100 % des cas, tandis qu’une thrombopénie constituait l’anomalie biologique la plus fréquente, observée dans 89 % des cas. L’infection a été considérée comme sévère dans 10 cas (52,6 %) et caractérisée principalement par une encéphalopathie (9 cas, 90 %). Ces 9 cas d’encéphalopathie avaient tous une IRM pathologique (œdème cérébral dans tous les cas, avec hémorragies pour deux). Quatre présentent des séquelles persistantes.

***Conclusions*** : La transmission verticale mère–enfant du CHIKV est fréquente quand la mère est virémique à l’accouchement et conduit alors une fois sur deux à une infection néonatale sévère. Le Chikungunya représente donc une situation à risque néonatal pour les femmes virémiques en couche. Ces résultats doivent être pris en considération par les cliniciens et les autorités de santé publique confrontés à une épidémie de Chikungunya.

***Mots clef*** : virus Chikungunya, arbovirus, vecteur, nouveau-né, césarienne, placenta, infection, encéphalopathie, IRM.
